# Development and Healing Process of Severe Radiodermatitis in Patients With Head and Neck Cancer Undergoing Radiotherapy: A Scoping Review

**DOI:** 10.1155/nrp/1940552

**Published:** 2024-12-31

**Authors:** Nao Miyamae, Yuko Imakata, Mao Kunimitsu, Makoto Oe

**Affiliations:** ^1^Department of Fundamental Nursing, School of Nursing, Hyogo Medical University, Kobe, Japan; ^2^Department of Adult Nursing, Ishikawa Prefectural Nursing University, Kahoku, Japan; ^3^Faculty of Health Sciences, Institute of Medical, Pharmaceutical and Health Sciences, Kanazawa University, Kanazawa, Japan

## Abstract

**Aims:** To summarize the morphological characteristics and development and healing processes of severe radiodermatitis for examining the factors contributing to the development of severe radiodermatitis in patients with head and neck cancer.

**Methods:** This scoping review was conducted in accordance with PRISMA extension for Scoping Reviews. Data were extracted from selected references describing detailed conditions of severe radiodermatitis in patients with head and neck cancer. The data were organized separately for radiotherapy, chemoradiotherapy, and bioradiotherapy.

**Data Sources:** Medline, PubMed, CINAHL, and Cochrane Central Register of Controlled Trials databases were used to search for papers from 2000 to December 2023.

**Results:** 11 out of 658 references met the criteria for this review. The morphological characteristics of severe radiodermatitis were categorized by symptoms, site, and shape, and a condition in which moist desquamations and associated crusts spreading to the anterior and lateral neck areas were extracted. In bioradiotherapy, the process of keratinocyte degeneration and formation of blisters under the epidermis leading to moist desquamations was extracted. In chemoradiotherapy, the process of epithelization was extracted 1 week following the occurrence of moist desquamations.

**Conclusions:** Moist desquamations are more likely to occur in severe radiodermatitis in patients with head and neck cancer. Since they can fuse and spread, preventative measures to mitigate spreading are important. However, there is insufficient information to examine the causes of widespread moist desquamations. For preventing moist desquamations and establishing care methods to heal moist desquamations, it may be necessary to identify the symptoms, site, and shape, including the color tone and depth, and healing process during their occurrence.

## 1. Introduction

Head and neck cancer is a term used to describe cancers of the oral cavity, larynx, nasopharynx, oropharynx, and hypopharynx that affect approximately 880,000 patients or 4.6% of all reported cancer cases [1]. Radiotherapy remains the treatment of choice as most head and neck cancers are squamous cell carcinomas, which are highly radiosensitive and allow for the preservation of morphologic function. Nursing care related to radiotherapy for patients with head and neck cancer is highly necessary.

Radiodermatitis, one of the adverse events of radiotherapy, is caused by radiation-induced damage to the epidermis, dermis, and endothelial cells. Damage to epidermal basal cells and hair follicles makes the skin less regenerative and vulnerable. The dermis and vascular endothelial cells also undergo edema and vasodilation due to the inflammatory response. Radiodermatitis is radiation dose-dependent, with typical symptoms of erythema above 20 Gy, dry desquamation above 30 Gy, and moist desquamation above 40 Gy [2, 3]. The severity of radiodermatitis is generally assessed by The Common Terminology Criteria for Adverse Events (CTCAE), and Grade ≥ 3, which shows extensive moist desquamation, is considered severe radiodermatitis (Table 1). Severe radiodermatitis is very painful owing to moist desquamations and affects the quality of life of patients [4]; therefore, prevention of infections and early healing is important.

Radiodermatitis occurs in most patients owing to radiosensitization effects caused by the high doses (∼70 Gy) used during treatment in combination with chemotherapeutic agents such as cisplatin and cetuximab [5]. In addition to these radiotherapy-related factors, endogenous factors, such as age, smoking, body mass index, wrinkles, sun exposure, diabetes mellitus, and low nutritional condition before the initiation of treatment, contribute to the development of radiodermatitis [2, 6, 7]. The neck region is susceptible to severe radiodermatitis characterized by moist desquamations, wherein damage occurs in the sensitive part of the skin owing to radiodermatitis caused by mechanical irritation from body movement and clothing [8]. Moreover, in bioradiotherapy (BRT) with cetuximab, antiepidermal growth factor receptor (EGFR) antibodies impair the proliferation of keratinocytes that make up the skin, resulting in epidermal thinning and severe skin dryness, which, combined with radiation-induced skin damage, can cause severe radiodermatitis, characterized by moist desquamation and bleeding [9, 10].

Furthermore, the stratum corneum that has fallen off owing to skin dryness mixes with exudates and leads to crusting, which inhibits healing and increases the risk of infection [11]. Hence, patients with head and neck cancer are more likely to develop severe radiodermatitis, which may delay healing.

Although several studies have been conducted on the prevention of severe radiodermatitis in patients with head and neck cancer, a uniform care method remains lacking, and approximately 25% of patients who develop head and neck cancer have severe radiodermatitis [2, 7]. Strategies to prevent severe radiodermatitis include preventing radiodermatitis itself and radiodermatitis from becoming more severe if occurs. Regarding the former, aside from avoiding mechanical irritation, the washing and moisturizing recommended as preventive care for general radiodermatitis have been applied to patients with head and neck cancer. In studies that compared the severity of radiodermatitis after applying Olive oil [12]. For trolamine emulsion [13] and biafine [14] from the start of radiotherapy, there was no difference in the incidence of Grades 1–2 radiodermatitis with the control group. Although there was a lower incidence of Grade 3 radiodermatitis than the controls in all included studies, the incidence ranged from 6.4% to 20%, indicating that radiodermatitis could not be prevented. Aside from the continued avoidance of mechanical irritation after the latter radiodermatitis, maintenance of the skin's condition, such as cleaning and moisturizing, is selected based on the grade [15]. In a study on managing radiodermatitis in patients with head and neck cancer, gentle cleaning was performed for Grade 1 radiodermatitis, and a moist environment was maintained in addition to cleaning for Grade 2 radiodermatitis; the results indicated that the incidence of Grades 2 and 3 radiodermatitis was 56.0% and 9.7%, respectively [16]. These results imply that the aforementioned care methods do not prevent severe radiodermatitis. British Columbia Cancer Agency (BCCA) guidelines recommend maintaining a moist environment to reduce inflammation for Grade 1 radiodermatitis and preventing infection for Grades 2-3 [17]. Although cleansing and moisturizing, which are care methods used to prevent radiodermatitis, are effective in preventing skin dryness and loss of the skin barrier function, it is difficult to completely prevent moist desquamations caused by the thinning and shedding of the epidermis. Moreover, although the care methods used to maintain a moist environment following radiodermatitis are intended to heal moist desquamations, cases of the epidermis not forming and moist desquamations expanding while the basal cell functions are not restored have been reported. Therefore, the currently recommended care methods are not sufficient to prevent severe radiodermatitis in patients with head and neck cancer, and the preventive care that should be provided remains to be determined.

Prevention and early healing of severe radiodermatitis in patients with head and neck cancer requires care methods that correspond to its development, healing process, and factors involved. One method is to identify the morphological characteristics of severe radiodermatitis from its development to healing and identify the factors that influence these characteristics. For example, in a study that revealed the developmental factors from the morphological characteristics of dermatitis surrounding breast cancer wounds, the presence of a “radial shape matching the dressing” indicated that the skin damage was caused by exudates, and in cases of severe exudates, the care method involves increasing the frequency of changing dressings and using highly absorbent dressing materials [18]. In this review, the morphological characteristics of severe radiodermatitis were extracted from the literature to discuss its occurrence. This will help to facilitate care methods for the prevention and early healing of severe radiation dermatitis in patients with head and neck cancer.

### 1.1. Review Question

The review question was “What are the morphological characteristics of the development and healing process of severe radiodermatitis in patients with head and neck cancer?”

### 1.2. Objective

To summarize the morphological characteristics of severe radiodermatitis from the references, focusing on its symptoms, shape, and site, and also summarize the development and healing process of severe radiodermatitis to examine the factors contributing to its development in patients with head and neck cancer.

Patient, Concept, and Context (PCC) was defined according to the JBI Manual [19] as follows: A Patient was an adult undergoing radiotherapy alone (RT) for head and neck cancer; the Concept was severe radiodermatitis (Grade ≥ 3), and the Context was an acute adverse event within 3 months of treatment completion.

## 2. Methods

### 2.1. Protocol

No review protocols were registered. This scoping review was conducted in accordance with the guidelines for scoping review reports (PRISMA-ScR: PRISMA extension for Scoping Reviews) [20].

### 2.2. Selection Criteria

The search was limited to papers written in English published between 2000 and December 2023. Intensity modulated radiation therapy (IMRT), a high-precision RT, has been implemented since 2000, at which point adverse events, such as radiodermatitis, have decreased; hence, publications after 2000 were selected.

Severe radiodermatitis is defined as Grade ≥ 3 or higher or severe in the Radiodermatitis Assessment Tool or determined to be severe radiation dermatitis by the authors of the selected publication.

The selection criteria were as follows: (1) studies on radiotherapy in adult patients with head and neck cancer, (2) studies with specific descriptions of severe (Grade ≥ 3) radiodermatitis symptoms, (3) studies on acute radiodermatitis (within 3 months of treatment completion), and (4) original papers or case reports. The exclusion criteria were as follows: (1) studies on diagnosed skin diseases other than radiodermatitis, (2) studies related to recall phenomenon, (3) studies related to re-irradiation, and (4) studies on non-X-ray therapy (such as proton beam or heavy particle beam).

This review was not limited to combination chemotherapy or cancer type; studies that focused on adults who developed severe radiodermatitis due to radiotherapy for head and neck cancer were also selected. The most crucial selection criterion was the presence of a description of the specific symptoms of severe radiodermatitis.

### 2.3. Information Sources

Searches were conducted using the Medline, PubMed, CINAHL, and Cochrane Central Register of Controlled Trials databases.

### 2.4. Search

The search was conducted using “AND” with head and neck cancer, radiodermatitis, and severe disease as the primary search concepts. The search formula used was (radiodermatitis OR radiation dermatitis OR skin reaction) AND (head and neck cancer OR head and neck neoplasms OR squamous cell carcinoma of head and neck) AND (severe OR grade 3 OR infection).

### 2.5. Reference Selection

Two authors (Miyamae and Imakata or Kunimitsu) independently screened the articles for titles and abstracts and excluded those that did not meet the inclusion criteria. Articles deemed suitable were read in their entirety by two authors (Miyamae and Imakata or Kunimitsu) and screened according to the selection criteria; articles that did not include specific symptoms of severe radiodermatitis were excluded. Disagreements regarding the selected studies were discussed.

### 2.6. Data Extraction Process

To determine the variables to extract, a data chart form was created by one author (Miyamae). The data were extracted by one author (Miyamae) and validated by two authors (Imakata and Oe). Discrepancies in the extracted data were discussed and resolved by the three authors.

### 2.7. Data Items

Since BRT has different effects on the skin depending on the anti-EGFR antibody, the following data were organized separately for RT, chemoradiotherapy (CRT), and BRT: (a) study authors/year of publication/country, (b) aims, (c) study design/participants, (d) total dose/combination drags, (e) radiodermatitis evaluation tool, (f) morphological characteristics of severe radiodermatitis (symptoms, shape, and site), and (g) developmental and healing process.

### 2.8. Integration of Results

The results were integrated through discussions among the researchers according to the JBI Manual [19].

## 3. Results

### 3.1. Selection of References

**Figure 1:**
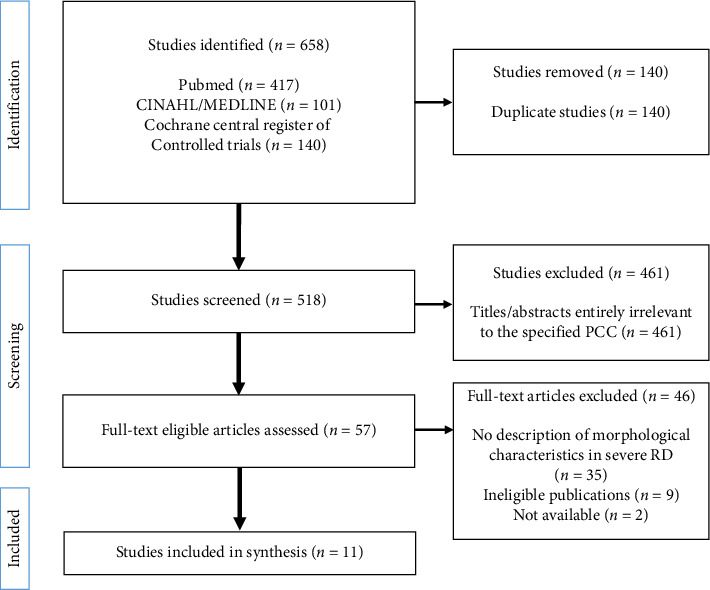
PRISMA-ScR flow diagram [19].

The initial search yielded 658 publications, and after excluding duplicates (*n* = 140), 518 references were screened, of which, based on the title and abstract, 461 were excluded. The entire text of the remaining 57 articles was read, of which 46 were excluded. A total of 35 articles did not indicate the symptoms of severe radiodermatitis, 9 did not match the publication type, and 2 were excluded owing to unavailability. A total of 11 articles were finally included in the analysis. The PRISMA-ScR flow diagram for reference selection is shown in Figure 1.

### 3.2. Characteristics of the Included Articles

The selected articles were studies conducted in different countries, from 2008 to 2020. Of the 11 studies, 6 were original papers and 5 were case reports; only 1 aimed to identify the symptoms of severe radiodermatitis. The types of radiation therapy administered to the subjects were RT for 1 patient, CRT for 4 patients, and BRT for 6 patients. The characteristics of the articles and data extracted are listed in Table 2.

### 3.3. Morphological Features Associated With Severe Radiodermatitis

The morphological features of severe radiodermatitis were classified into symptoms, shape, and site.

#### 3.3.1. Symptoms

The symptoms were extracted from all included articles. Moist desquamation and bleeding were extracted from the RT reference [21]. Moist desquamation [13, 22–24], ulcerative moist desquamation [22], and edema [13, 22] were extracted from 4 CRT references. Skin necrosis [25], erythematous rash around moist desquamations [26], erythematous papules [27], pustules [27], bleeding erosions covered with crusts [27], moist desquamation [28], skin necrosis with thick hemorrhagic crusts [28], erythema [29], crusts [29], and hemorrhagic crusts [30] were extracted from 6 BRT articles. The main symptoms were moist desquamation and associated exudates and bleeding that turned into crusts.

#### 3.3.2. Shape

Shapes were extracted from 6 articles. Moist desquamation spread evenly (to the extent that skin folds were unclear) [21] was extracted from the RT article. Patchy moist desquamation [24] and confluent moist desquamation other than in skin folds [13, 22, 24] were extracted from 3 CRT articles. Confluent erythematous papules [27] and extensively confluent hemorrhagic crusts [27] were extracted from 2 BRT articles.

#### 3.3.3. Sites

Sites were extracted from 4 articles. Anterior and bilateral neck [23] were extracted from 1 CRT article; the side of the neck [25], upper back [25], upper chest [25], lower half of face [25, 26], upper neck [26], face [27], and neck [27] were extracted from 3 BRT articles.

### 3.4. Developmental and Healing Process

The developmental process was extracted from 1 article. Erosion at 40 Gy and ulceration at 58 Gy were extracted in BRT. This article also described the histopathologic developmental process, which resulted in acute cytotoxic dermatitis with vacuolic degeneration of basal keratinocytes and a subepidermal blister formation leading to a complete loss of the epidermis.

The healing process was extracted from 2 articles. The healing process after the development of moist desquamation in CRT, proliferative phase after 6 days, healing phase after 12 days, and completely healed phase after 18 days were also extracted [23]. In another CRT article, re-epithelialization after 3 days, healing of the moist wound after 5 days, and complete recovery after 7 days were extracted [22].

### 3.5. Integration of Results

A morphologic characteristic of severe radiodermatitis in patients with head and neck cancer was the even spread of moist desquamation in RT. In CRT, the patient had moist desquamation on the anterior and bilateral neck, with patchy moist desquamation and confluent moist desquamation other than in skin folds. In BRT, erythematous papules confluent to the face and neck, bleeding, skin necrosis on the side of the neck and upper back, and extensively confluent hemorrhagic crusts had occurred. More diverse symptoms and sites were extracted in BRT compared with those in CRT. However, morphological characteristics other than symptoms, shape, and site, such as color tone and depth, were not extracted.

In BRT, ulceration, subepidermal blister formation, and finally complete loss of the epidermis and degeneration of keratinocytes were involved in the development of moist desquamation. The healing process involving a tendency to epithelialize 1 week after the development of moist desquamation followed by complete healing was extracted in CRT. However, no information on the developmental process in CRT or the healing process in BRT was extracted.

## 4. Discussion

This scoping review focused on the morphological characteristics of severe radiodermatitis in patients with head and neck cancer, such as symptoms, shape, and site, as well as the development and healing process of severe radiodermatitis. Morphological characteristics were also summarized separately for RT, CRT, and BRT. To the best of our knowledge, this is the first review that focused on the morphological characteristics of severe radiodermatitis in patients with head and neck cancer.

### 4.1. Morphological Characteristics and Factors Involved in the Development of Severe Radiodermatitis in Patients With Head and Neck Cancer

In RT and CRT, moist desquamation confluent to the anterior and bilateral neck areas occurs. High doses of ∼70 Gy are used in radiotherapy for head and neck cancer, as well as for the skin surface, triggering apoptosis in the epidermal cells and causing them to fall off, which can lead to moist desquamation [3, 31]. Moreover, the anterior and bilateral neck areas are more prone to moist desquamation because the neck has a wide range of motion and is prone to mechanical irritation by wrinkles and clothing. Symptoms characteristics of BRT were crusting and bleeding. BRT uses anti-EGFR antibodies in conjunction with RT, and their synergistic effects can cause premature skin dryness and skin necrosis [10]. It has also been revealed to produce numerous inflammatory exudates, exacerbating inflammation, and accompanied by crust formation that increases pain, bleeding, and risk of infection [10, 11]. This suggests that crusting and bleeding extracted as BRT symptoms were the result of moist desquamation. The moist desquamations are prone to occur and can fuse and spread; this was common in RT, CRT, and BRT of severe radiodermatitis in patients with head and neck cancer. Furthermore, CRT and BRT have different morphological characteristics. However, data on the site and shape to examine the factors causing the occurrence are lacking and so are data on color tone or depth.

The process of moist desquamation development in BRT involves structural changes in the skin, such as the degeneration of keratinocytes and subepidermal blister formation. These results suggest that radiation and chemotherapy alter the skin structure, making it more prone to moist desquamation caused by mechanical irritation. Moreover, in CRT, epithelization occurs approximately 1 week following the development of moist desquamation. However, the detailed process of how severe radiodermatitis develops and heals remains to be determined, and the reasons for the development of severe radiodermatitis remain unclear.

### 4.2. Clinical Application

One suggestion for clinical practice is to implement care methods for patients with head and neck cancer undergoing radiotherapy to prevent the spread of moist desquamation. Particular attention should be given to mechanical irritation, such as friction since moist desquamations are prone to enlarge in BRT. Care methods that maintain a moist environment may be appropriate when moist desquamations are epithelializing with no infection. If no healing trend is observed within a week or so after moist desquamations develop in CRT, the condition of moist desquamations should be monitored, and appropriate care methods should be selected.

### 4.3. Future Prospects

We identified the important perspectives for reviewing preventive care for severe radiodermatitis in patients with head and neck cancer. In severe radiodermatitis in patients with head and neck cancer, moist desquamations and associated exudates and bleeding had spread to form crusts. However, how moist desquamations expand and heal remains to be determined. Data on symptoms, site, and shape, including coloration and depth observed in the process of the occurrence of moist desquamation, are needed to better examine the causes of moist desquamation. Moreover, identifying the healing process will allow for the consideration of care methods for the early healing of moist desquamations.

### 4.4. Limitations

This study has several limitations. First, the keywords “severe,” “Grade 3,” or “infection,” were used to summarize the morphological characteristics of severe radiodermatitis in head and neck cancer; however, some publications may not have been extracted using these keywords. Moreover, since only articles written in English were included, relevant references in other languages were not extracted.

## 5. Conclusions

In this scoping review, we summarized the morphological characteristics and development and healing process based on the symptoms, shape, and site in RT, CRT, and BRT to examine factors contributing to the development of radiodermatitis in head and neck cancer patients.

The morphological characteristics of severe radiodermatitis in patients with head and neck cancer were moist desquamations and associated exudates and bleeding that spread to form crusts. The occurrence site was divided into anterior and bilateral neck areas. In BRT, the process of keratinocyte degeneration and subepidermal blister formation leading to moist desquamation was revealed, while in CRT, epithelization as part of the healing process occurred 1 week following the development of moist desquamation.

This scoping review identified that moist desquamation should be considered when developing a strategy for the care of severe radiodermatitis in patients with head and neck cancer. The prevention of moist desquamation and care needed to heal them can be achieved by identifying the symptoms, site, shape, including coloration and depth, and healing process during the development of moist desquamation.

## Figures and Tables

**Table 1:** CTCAE version4 radiodermatitis evaluation criteria.

Grade 1	Grade 2	Grade 3	Grade 4
Follicular, faint or dull erythema/epilation/dry desquamation/decreased sweating	Tender or bright erythema and patchy moist desquamation/moderate edema	Confluent, moist desquamation other than skin folds, and pitting edema	Ulceration, hemorrhage, necrosis

Abbreviation: CTCAE, Common Terminology Criteria for Adverse Events.

**Table 2:** Characteristics of the study and data on severe radiodermatitis.

Method	Study author/year/country	Aims	Study design/participants	Total dose/combination drags	RD evaluation tool	Morphological characteristics of severe RD			Developmental and healing processes
						Symptoms	Shape	Site	
RT	Zenda S, et. al/2016/Japan	To develop a grading atlas of radiation dermatitis in Japanese head and neck cancer patients	Prospective picture collection study/receive definitive radiotherapy or chemoradiotherapy (*N* = 118), 38 photographs were selected in 1600 photographs	50 Gy/radiation only	Common Terminology Criteria for Adverse Events (CTCAE) v4.03	Moist desquamation, bleeding induced by minor trauma or abrasion immediately after removal of the gauze coating.	Moist desquamation was spread evenly (to the extent that skin folds became unclear)	Not described	Not described
CRT	Sahu S, et al./2020/India	Not described	Case report/a 58 year old man was diagnosed as a case of carcinoma of larynx with bilateral neck nodes (stage T4a N2 c M0; AJCC 7th Edition).	76 Gy/cisplatin	The radiation oncology toxicity grading (RTOG) scale Bates-Jensen wound assessment tool	Moist desquamation	Not described	The anterior and bilateral neck	Healing process Day-6: bio-burden with proliferative phase Day-12: healing phase Day-18: complete healed phase
	Yan J, et al./2020/China	To compare the effect of mepitel film and biafine cream on (1) overall skin reaction severity and (2) on the rates of moist desquamation	A randomized, intra-patient controlled open-label stage II clinical trial comparing the effects of mepitel film against those of biafine cream on the severity of acute radiation-induced skin reactions/a total of 39 patients	70–74 Gy/nedaplatin	Radiation-induced skin reaction assessment scale (RISRAS) and the expanded RTOG scale	Moist desquamation	Patchy moist desquamation confluent moist desquamation other than in skin folds	Not described	Not described
	Lee J, et al./2016/South korea	To investigate the effects of rhEGF on the healing process in head and neck cancer patients with radiation-induced dermatitis	Observational study/seven patients, including three with oropharyngeal, two with nasopharyngeal, and one each with hypopharyngeal and laryngeal carcinoma	70 Gy/six of the seven patients received concurrent chemotherapy	The common toxicity criteria (CTC) grading system	An ulcerative moist desquamation moist desquamation, pitting edema	Confluent moist desquamation > 1.5 cm diameter and not confined to skin folds	Not described	Healing process Day-3: disappearance of exduced, reepithelialisation Day- 5:the moist wound had healed Day-7: completely recovered
	Abbas H, et al./2012/Egypt	To test trolamine compared with the usual supportive care for patients with head and neck cancer undergoing radiation therapy with concurrent chemotherapy	This phase III trial was designed to test trolamine emulsion compared with the usual supportive care. Treatment group: 15 cases; control group: 15 cases	≥ 66 Gy/Cisplatin	RTOG scale	Moist desquamation, pitting edema	Confluent moist desquamation (other than skin folds)	Not described	Not described
BRT	Koutcher L, et al./2009/USA	To determine the prevalence of serious (≧ Grade 3) radiation dermatitis in patients with locally advanced HNC treated with concurrent cetuximab and radiation to describe the medical history, clinical presentation, and dermatitis manifestation of the patients who developed Grade 4 dermatitis	Retrospective chart review/patients with HNC treated with concurrent radiation (*N* = 115)	Not described/cetuximab	CTCAE v3.0	Spontaneous cutaneous bleeding, skin necrosis	Not described	The side of the neck, the upper back, and the upper chest, lower face, and bilateral neck	Not described
	Azad A./2009/Australia	Not described	Case report/a 69-year-old Caucasian male	66 Gy/cetuximab	Not described	A moist desquamation circumferential erythematous rash	Not described	The lower half of face and upper neck	Not described
	Vano-Galvan S, et al./2008/Spain	Not described	Case report/a 77-year-old man with squamous-cell carcinoma of the oropharynx	70 Gy/cetuximab	Not described	Erythematous papules, pustules, bleeding erosions covered by crusts	Confluent erythematous papules	The face and neck, more marked on the irradiated field	
	Bölke E, et al./2008/Germany	To provide detailed information on the developed a severe radiation dermatitis during cetuximab plus radiation therapy	Case report/two cases of HNC patients	66–72 Gy/cetuximab	CTCAE v3.0	An erosive dermatitis, skin necrosis with thick hemorrhagic crusts	Not described	Not described	Developmental process 40 Gy: an erosive 58 Gy: an ulcerative Histopathology: an acute cytotoxic dermatitis with vacuolic degeneration of basal keratinocytes and a subepidermal blister formation leading to a complete loss of the epidermis.
	Presta G, et al./2019/Switzerland	To report our experience with using hyaluronic acid gel to heal a severe skin erythema and its efficacy	Case report/a 77 year old man	70 Gy/cetuximab	RTOG scale	Erythema, dry, desquamation, crust	Not described	Not described	Not described
	Bonomo P, et al./2019/Italy	To assess the impact of an inhouse skin policy protocol on the tolerability of concurrent RT and cetuximab in patients with head and neck squamous cell carcinoma	Cohort study/51 patients were treated with RT and cetuximab in patients with head and neck squamous cell carcinoma. No management: 26 patients advanced wound care: 25 patients	66–70 Gy/cetuximab	CTCAE v.4.1 BRD severity was graded according to Bernier's	Hemorrhagic crusts	Extensive confluent hemorrhagic crusts	Not described	Not described

Abbreviations: BRT, bioradiotherapy; BRD, bioradiodermatitis; CRT, chemoradiotherapy; RD, radiodermatitis; RT, radiotherapy alone.

## Data Availability

Data sharing is not applicable to this article as no new data were created or analyzed in this study.
